# Sexual Allometric Monomorphism in Araucan Pig from Colombia: Preliminary Results

**DOI:** 10.3390/ani10101763

**Published:** 2020-09-28

**Authors:** Arcesio Salamanca-Carreño, Jordi Jordana-Vidal, René Alejandro Crosby-Granados, José Norberto Arias-Landazábal, Pere M. Parés-Casanova

**Affiliations:** 1 Grupo de Investigaciones los Araucos, Facultad de Medicina Veterinaria y Zootecnia, Universidad Cooperativa de Colombia, 810001 Arauca, Colombia; asaca_65@yahoo.es (A.S.-C.); racrosbyg@yahoo.com (R.A.C.-G.); arias.landazabal@campusucc.edu.co (J.N.A.-L.); 2 Departament de Ciència Animal i dels Aliments, Universitat Autònoma de Barcelona, 08193 Bellaterra, Catalonia, Spain; jordi.jordana@uab.es; 3 Departament de Ciència Animal, ETSEA, Universitat de Lleida, 25198 Lleida, Catalonia, Spain

**Keywords:** creole breeds, multivariate allometry, relative growth, size sexual dimorphism, sexual selection

## Abstract

**Simple Summary:**

Breed morphological profiling is the first step towards the conservation of local genetic resources, and data obtained from different approaches improve knowledge on them. Morphometric measurements performed on animals are important tools in the assessment of growth and body development as they make it possible to perform quantitative analysis. This study aimed to evaluate the allometric growth of the Araucan pig breed, a creole breed from Arauca, East Colombia, locally known as “Sabaneros”. Little is known about the production, economic importance, and geographical distribution of these animals. Knowledge on the genetics, morphology, growth potential, and yield characteristics of a breed is essential to establish a rational production system. Results showed that in Araucan pigs, sexual differences do not increase with body size.

**Abstract:**

This study aimed to evaluate the allometric growth of the Araucan pig breed, a creole breed from Arauca, East Colombia, locally known as “Sabaneros”, in relation to different quantitative traits and considering genders separately. To do this, a total of 31 male and 27 female Araucan pigs, ranging from 4 to 48 months of age, were studied in order to evaluate their growth patterns, using a multivariate approach. Animals belonged to different farms (“fincas”) of the Department of Arauca, Colombia. From each individual, 10 quantitative traits were obtained: face width, croup height, croup length, croup width, tail base height, hock height, loin height, cannon length, and length and width of ear. Our results, which must be interpreted as preliminary, showed that the Araucan pig is allometrically monomorphic as sexual differences do not increase with body size. We suggest that although males and females have evidently different reproductive roles, during growth they shift the allocation of energy to structures linked to environmental adaptation rather than those linked to reproduction.

## 1. Introduction

Breed morphological profiling is the first step towards the conservation of local genetic resources, and data obtained from different approaches improve knowledge on them [1]. Morphometric measurements performed on animals are important tools in the assessment of growth and body development as they make it possible to perform quantitative analysis.

In animals, body measurements give significant information on morphological structure and development abilities, representing also the most influential factors on determining the most appropriate animals for a desired efficiency [2,3]. In animals, size contributes to body structure, harmony, and balance, but also to physiological characteristics and to mechanisms for adaptation to the environment [4,5].

Various physiological and pathological conditions, as well as different environmental and management conditions, can influence the size of the animals. Allometric growth studies examine the relative growth of a body component relative to a collection of other components, being an effective manner to study their development [6,7,8]. Morphometric measurements performed on animals provide additional information that is useful for determining phenotypic and genetic trends of growth of animals over the years [9]. Broadly, allometry has been suggested as having an influential impact on sexual shape dimorphism [10].

Iberoamerica has a great number of animals completely adapted to local conditions whose production is sustainable and ecological in all its phases, and whose product has a high nutritional value [11]. Known as “Creoles”, the pigs were not introduced to Colombia until the 16th century [9,12]. There they underwent hundreds of years of natural selection on the basis of adaptation to local environmental characteristics [13,14], which included survival and reproduction in geographical areas of tropical climates [11].

Over the past few decades, awareness has been raised about the importance of preserving animal genetic resources, although research initiatives are not yet totally widespread to most of the American continent [11,15]. This study aimed to evaluate the allometric growth of the Araucan pig breed, a local Colombian breed, in relation to different quantitative traits and considering genders separately. To the authors’ knowledge, this is the first time this topic has been analyzed for this population, although it has been done for other Creole pigs [9].

Araucan pigs are believed to have descended from pigs first brought from Spain during the 15th century and were selected for adaptation to the local conditions [14,15,16]. Locally known as “Sabaneros”, they are managed under extensive conditions in the Araucan plains of East Colombia, receiving scanty care and thriving semi-wildly on the savannah. Pigs are permanently exposed to environmental disturbances and irregular food supply. Little is known about their production, economic importance, and exact geographical distribution, which deserves special attention from the scientific community. The comprehension of this extensive production system involves the conservation of the environment and respect and welfare of the animals and also the farmer (“llanero”).

## 2. Materials and Methods

### 2.1. Area of Study

**Figure 1:**
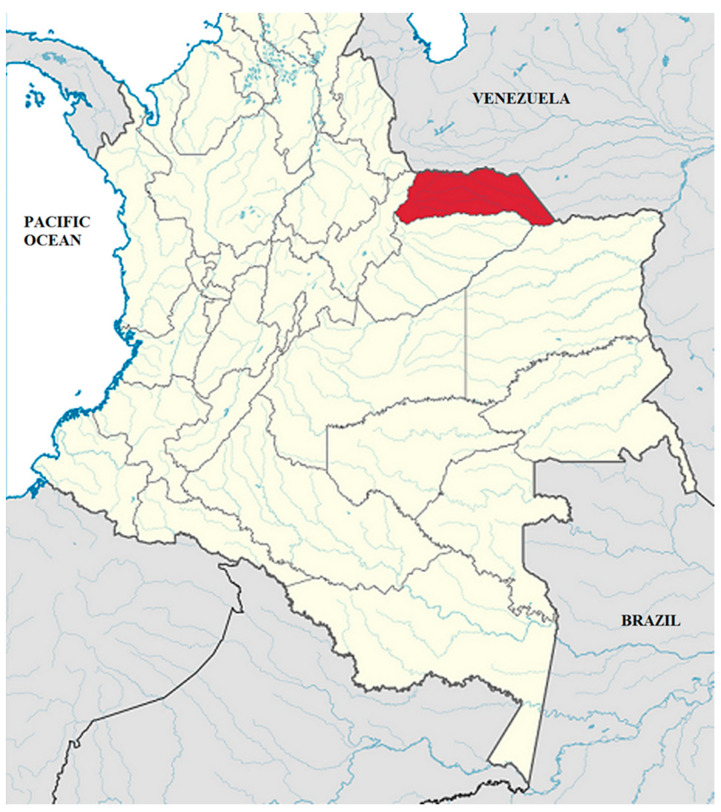
Arauca department located in the “Llanos Orientales” of Colombia.

Arauca is a department of Colombia located on the Orinoco Basin of Colombia (the “Llanos Orientales”) in the extreme east, bordering Venezuela. Its territory covers an area of 23,818 km^2^. It is an ecosystem that is characterized by a plateau (Figure 1). The region is located between the Arauca and Casanare rivers. Seventy-five percent of the land is flat. Over the year, the temperature typically varies from 72 °F to 97 °F and is rarely below 69 °F or above 102 °F. This huge area receives low touristic impact, and waste dumped by multinational companies is its main threat.

### 2.2. Sample

A total of 31 male and 27 female Araucan pigs, ranging from 4 to 48 months of age, were studied. Male and female pigs were not subjected to castration. Animals belonged to different farms (“fincas”) of the Department of Arauca, East Colombia, and were managed in the extensive traditional way. Environmental conditions and possible management differences between herds were identical. From each individual, 10 quantitative traits were obtained: face width, croup height, croup length, croup width, tail base height, hock height, loin height, cannon length, and length and width of ear, following standard procedures [2,17,18].

### 2.3. Allometry

Allometry was evaluated using Jolicoeur’s multivariate allometric coefficient. Jolicoeur’s coefficient represents the coefficient (loading) of each morphometric variable for first axis of principal component analysis (PCA) divided by 1/(√N), where N is the number of variables, or appropriately quoted [19]. PCA was performed from a variance–covariance matrix. The first axis of the PCA contains most of the variability, and if all coefficients (loadings) of the morphometric variables for this axis are positive, it can be interpreted as a generalized body size. In order to test whether dimorphism increases with body size, a canonical discriminant analysis was performed to obtain the scores of individuals of the two sexes in a discriminant axis. Afterwards, following similar works [20], an ANCOVA based on these scores was used to test the differences between sexes and the interaction with the generalized body size (first PCA axis). Statistical analysis was done with PAST v. 2.17c software [21]. *p* values less than 0.05 were considered as statistically significant.

## 3. Results

**Figure 2:**
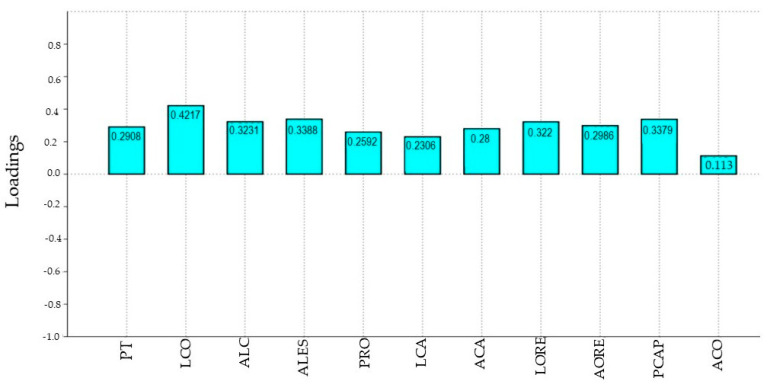
Coefficients (loadings) of the morphometric variables for first principal component, which explained 85.47% of the variability in individuals of both sexes of Araucan pig (*n* = 58). As all loadings were positive, they can be interpreted as a generalized body size. Thoracic circumference (PT), body length (LCO), withers height (ALC), sternum height (ALES), head length (LCA) and width (ACA), ear length (LORE) and width (AORE), hindlimb cannon perimeter (PCAP), knee circumference (PRO), and hock height (ACO).

**Figure 3:**
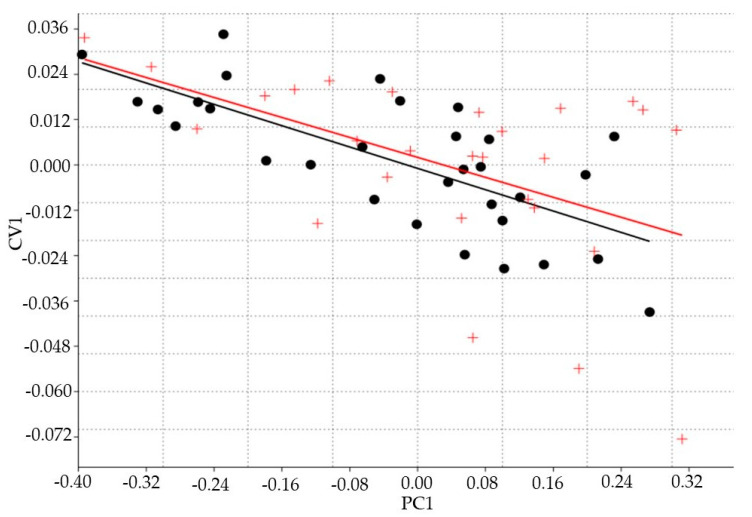
Relationship between the scores of canonical variate analysis (CV1) and the generalized size (first principal component PC1) in individuals of both sexes of Araucan pig (31 males, ●, and 27 females, +). There were no statistical differences between genders (*p* = 0.534).

The first PCA axis, which represented the generalized size as all loadings appeared positive (Figure 2), explained 85.47% of the variability. Results of the ANCOVA showed an interaction between the morphometric differentiation of the sexes (first axis of discriminant function) and the generalized body size (first PCA axis). This result demonstrated that the morphometric traits increased with increasing age (Table 1) with no statistical differences between genders (*p* = 0.534) (Figure 3).

## 4. Discussion

Morphometry can help in the characterization of animal breeds as well as the definition of their use [2]. These measurements are influenced by the development of bones as well as deposition of muscle and fat. Measurements that are mainly linked to bone growth include face width and cannon length, as well as hock height. Measurements linked to soft tissue (muscle and fat) deposition are croup and loin heights.

Our preliminary results showed that sexual dimorphism of Araucan pigs does not increase with body size (i.e., they show a monomorphic allometry). In the early stages of development, males and females are more similar than in the late stages, at least in the studied range (4 to 48 months). We suggest that although males and females have different reproductive roles, during growth both shift the allocation of energy to structures more related to adaptation than to reproduction. Thus, in late stages (48 months), the individuals of the two sexes are morphometrically similar. An investment of males in increasing size would be only needed if it would provide advantages during fights with other males, and possibly during courtship and mating, but as herds are maintained in open herds, they remain very isolated from neighbor groups and territoriality is not necessary. Similar allometries for both genders within a breed, but differing between breeds, have been obtained by other authors for other swine creole breeds [9].

As the sample size is relatively scarce and unbalanced according to the sex and age groups, there is a major flaw in the experimental design, so the results cannot be conclusive and need to be interpreted as a preliminary study. Anyway, our preliminary results should be taken into account when developing management and conservation programs for the breed.

## Figures and Tables

**Table 1:** ANCOVA of the scores for male (*n* = 31) and female (*n* = 27) individuals and age of Araucan pigs, using the generalized body size (first axis of the principal component analysis, PC1) as a covariate.

Effect	Sum of Squares	Degrees of Freedom	Mean Squares	F	*p*
Sex	0.0550	1	0.05500	0.9831	0.0397
Age	1.2976	7	0.18537	3.3132	0.0001
Sex * Age	−1.7422	7	−0.24889	−4.4485	0.4627
Residual	2.3499	42	0.05594		

* sex by age interaction.
